# Supplementary material for: Overview of the ESCAPE Dark Matter Test Science Project for astronomers
Source: Open Res Eur. 2026 Apr 9;5:336. Originally published 2025 Nov 6. [Version 3] doi: 10.12688/openreseurope.21610.3 (PMC12775671; doi:10.12688/openreseurope.21610.3)
Supplement: Peer review history

# Reviewer report: Reviewer response for version 3

**DOI:** 10.21956/openreseurope.25376.r72100

**Published:** 2026-04-27

**Authors:** Martine Lokken

**Affiliations:** 1 Institut de Física d’Altes Energies (IFAE), Barcelona, Spain

I am happy with the updates to the article and believe them to be sufficient to approve the indexing.

Is the review written in accessible language?

Yes

Are all factual statements correct and adequately supported by citations?

Partly

Are the conclusions drawn appropriate in the context of the current research literature?

Yes

Is the topic of the review discussed comprehensively in the context of the current literature?

Partly

Reviewer Expertise:

Cosmology, astrophysics, large-scale structure

I confirm that I have read this submission and believe that I have an appropriate level of expertise to confirm that it is of an acceptable scientific standard.

# Reviewer report: Reviewer response for version 3

**DOI:** 10.21956/openreseurope.25376.r72099

**Published:** 2026-04-16

**Authors:** Eugene Oks

**Affiliations:** 1 Auburn University, Auburn, AL, USA

According to Abdurashidova et al. (2022, *Astrophysical Journal* 924, 51), the latest observations from the Hydrogen Epoch of Reionization Array (HERA) do not disprove the results from EDGES (2018). Pearson et al. appear to have overlooked this and have continued attempts to question the observational results from EDGES (2018). Therefore, I do not recommend the paper for indexing.

Is the review written in accessible language?

Yes

Are all factual statements correct and adequately supported by citations?

Partly

Are the conclusions drawn appropriate in the context of the current research literature?

Yes

Is the topic of the review discussed comprehensively in the context of the current literature?

Partly

Reviewer Expertise:

Astrophysics; Particle Physics

I confirm that I have read this submission and believe that I have an appropriate level of expertise to state that I do not consider it to be of an acceptable scientific standard, for reasons outlined above.

# Reviewer report: Reviewer response for version 2

**DOI:** 10.21956/openreseurope.23894.r65953

**Published:** 2026-01-06

**Authors:** Martine Lokken

**Affiliations:** 1 Institut de Física d’Altes Energies (IFAE), Barcelona, Spain

This article presents an introductory overview of the evidence for dark matter (DM), focusing on the current status of the cold dark matter model, and discusses several ongoing direct and indirect detection experiments in particle physics in the context of the ESCAPE DM Test Science Project. The authors then review several astrophysics efforts to further constrain the nature of DM, and present several available software tools for both fields on the Virtual Research Environment (VRE).

 The DM Test Science Project and associated VRE are presented in a clear way that can be useful to astronomers, as is one of the goals of the article. The article is scientifically accurate, but the discussion of baryonic dark matter could be misleading. Additionally, as one of the stated goals of the article is to increase collaboration across fields, the review would benefit from more explicit linking between the astrophysics and particle physics experimental goals and scientific products. As it stands, the article (particularly sections 2 and 3) includes fairly disjoint overviews of particular ongoing DM experiments in the particle physics and astrophysics fields, but lacks clear discussion of connections between those experiments. This is the key point that I believe should be addressed before full acceptance of the article. There are also some issues with clarity; I provide further details in the following paragraphs.

 The discussion of 'baryonic dark matter' is potentially misleading; as it stands, it implies that the 70% of unaccounted for baryons are in the form of MACHOs. I recommend clarifying that in fact most of these baryons are expected to exist in the intergalactic medium, which is difficult to observe. In fact, recent observations by fast radio burst dispersion measures have found baryon fractions consistent with the expected cosmic fraction (e.g., Yang et al 2022, arXiv: 2211.04058). Therefore, I recommend removing text that conflates the 'missing baryons' with dark matter. While I understand some of this text may have been added in response to a previous review, the statements about baryonic dark matter should be qualified more carefully: e.g. when discussing the unaccounted-for baryons, one could state that " *even if* some of the unaccounted for baryonic matter belonged to an unexpected component like MACHOS, the implied ratio is *at least*..." I also note that the values given yield a ratio of 7.1, so the quoted value of 7.6 may be confusing to readers; I recommend quoting '7-8' or stating with higher precision the numbers leading to that calculation.

 The organization of section 2.1 could be confusing to a non-particle-physics expert. The introduction of MET in 2.1 seems to set up further discussion of challenges with detection via MET in ATLAS in the following sections, but the subsequent section, 2.1.1, appears to discuss another detection method without mentioning MET again. Reorganization of the MET discussion, clearer sectioning, and/or the addition of linking sentences to connect and/or contrast subsequent paragraphs would help make this review more understandable to an astronomy audience.

 Section 3 would be strengthened by providing more explicit links between the astrophysical dark matter searches discussed (subhalo lensing, gamma-ray production from DM annihilation) with the particle physics experiments discussed in Section 2. For example, many astronomers are aware of the astrophysical consequences of WDM or SIDM vs CDM, but are less likely to understand which of the indirect and direct detection experiments ongoing in the particle physics world are helping to constrain these models. I encourage the authors to state clear and explicit links between the experiments and models discussed in section 2 and those in section 3.

 Lastly, I recommend adding summary text at the end of Section 4 summarizing why it would be worthwhile to incorporate astronomy software into the VRE, and clarifying the vision and purpose for a fully integrated and coherent dark matter research software environment. To demonstrate the vision, it could be useful to provide an example of an ideal future workflow: for example, after all software integration, will the future VRE allow a researcher to compute cosmology, large-scale structure, and particle physics predictions for a given DM model using, e.g., GAMBIT, and then subsequently compute and plot constraints, entirely within the VRE? Would this be a workflow that was not previously possible for the community?

 I include below several minor grammatical notes to help improve readability:

 Abstract: "complementary" --> "complementarity"

 Introduction:

 "Short" --> "shorter" [free-streaming length]

 Incomplete sentence: "However, given the above astrophysical problems with CDM and that no clear detections of WIMPs have yet been observed in particle physics experiments, with other theoretical options also being considered"

 1.2: Ris --> RIs

 2.1: "and infer their existence" --> "and inferring their existence"

 "invisible particles (such as neutrinos or dark matter) particles" --> remove one 'particles'

 Perhaps the "Data and software availability" section could also link to the VRE, although I recognize it is linked in the footnotes already.

Is the review written in accessible language?

Yes

Are all factual statements correct and adequately supported by citations?

Partly

Are the conclusions drawn appropriate in the context of the current research literature?

Yes

Is the topic of the review discussed comprehensively in the context of the current literature?

Partly

Reviewer Expertise:

Cosmology, astrophysics, large-scale structure

I confirm that I have read this submission and believe that I have an appropriate level of expertise to confirm that it is of an acceptable scientific standard, however I have significant reservations, as outlined above.

# Response

**Published:** 2026-03-16

**Authors:** James Pearson

We thank the reviewer for their insightful comments and suggested revisions. To address each point in order: 1. A subsection has been added to the end of Section 3 to address this, relating Sections 2 and 3 and pointing to two reviews providing more details on the subject. 2. After some thought, some of the text of the relevant paragraph was deemed unnecessary, so the text has been simplified with more of a focus on baryonic dark matter in the IGM rather than MACHOs. 3. For clarity, a linking sentence has been added before 2.1.1. 4. See point 1 above. 5. As suggested, a paragraph has been added clarifying the vision of the VRE software and an idealised workflow. 6. Grammatical notes: These have been updated in the text.

# Reviewer report: Reviewer response for version 2

**DOI:** 10.21956/openreseurope.23894.r64392

**Published:** 2025-12-31

**Authors:** Felix Kahlhoefer

**Affiliations:** 1 Institut für Astroteilchen Physik, Karlsruher Institut für Technologie (KIT), Eggenstein-Leopoldshafen, Germany

I enjoyed reading this review and could not agree more with its overall goals. I am strongly in favour of making data sets and analysis routines publicly available and believe that the ESCAPE Dark Matter Test Science Project is making a valuable contribution in this regard that deserves to be highlighted and advertised. While I believe the manuscript should be published eventually, I have a number of suggestions for how the manuscript can be improved further:

 - In its current state, the ESCAPE Dark Matter Test Science Project suffers from a lack of completeness, which limits its usefulness for the community. This is most obvious in the case of dark matter direct detection experiments, where most of the leading experiments (such as XENONnT, LZ and Panda-X) are missing. The article should comment on the reasons for this (which could be for example a lack of publicly available information, or a simple lack of personpower), why the DarkSide experiment was chosen specifically, and possible strategies to make the data base more complete. 

 - In my opinion, the main obstacle for a closer collaboration between particle physicists and astrophysicists is that in many cases astrophysics and particle physics do not probe the same properties of dark matter, making any connection model-dependent. For any astrophysical observation that only relies on the gravitational interactions of dark matter (which includes the free-streaming of warm dark matter), constraints from laboratory experiments are irrelevant. And even constraints on dark matter self-interactions cannot be connected to other particle physics observables in a model-independent way. Because of this, providing a database of constraints is not sufficient unless it is combined with a framework for calculating observables from fundamental models. This is where most conceptual work is required and where scientists are needed who understand both fields well enough to make these connections. From the article it does not become clear how ESCAPE aims to address this issue.

 - Along the same lines, I have the impression that GAMBIT is somewhat misrepresented as a tool for statistical inference. Most of the effort in the development of GAMBIT actual go into the development of a database of models and the calculations needed to connect them to experimental likelihoods in an efficient way. However, GAMBIT currently does not consider many scenarios of interest for the astrophysics community, simply because the predictions are prohibitively expensive to calculate (e.g. N-body simulations needed to interpret gravitational lensing data). It would be interesting to discuss possible strategies to address this issue (e.g. neural network emulators, simulation-based inference techniques ...).

 - The first referee report highlights yet another difficulty of collaboration between particle physicists and astrophysicists, which is that many of the latter are fundamentally opposed to the idea of new particles beyond the Standard Model. I therefore appreciate the effort of the authors to address also these parts of the community and to highlight the compelling case for non-baryonic dark matter. I think this part of the discussion could be sharpened further by reminding the reader that even if we didn't have the ability to detect neutrinos on Earth, we would still be convinced of their existence through cosmological and astrophysical observations (e.g. the measurement of N_eff or the mechanism of supernova explosions). 

 - I find the recently added discussion of EDGES to be out of place. Of course it could be interesting to list various experimental/observational anomalies that could be related to the nature of dark matter (DAMA annual modulation, Galactic centre excess, keV x-ray/gamma-ray lines, diversity of dwarf galaxies...), many of which are more robust than the EDGES signal. But this is clearly not the goal of the article and would distract from its main message. Nevertheless, I do believe that 21cm intensity mapping is among the most interesting examples of the interplay between particle physics and cosmology and deserves to be mentioned in some form. Maybe the general concept can be discussed in a way that mentioned EDGES less prominently.

Is the review written in accessible language?

Yes

Are all factual statements correct and adequately supported by citations?

Yes

Are the conclusions drawn appropriate in the context of the current research literature?

Yes

Is the topic of the review discussed comprehensively in the context of the current literature?

Partly

Reviewer Expertise:

Dark matter, particle physics

I confirm that I have read this submission and believe that I have an appropriate level of expertise to confirm that it is of an acceptable scientific standard, however I have significant reservations, as outlined above.

# Response

**Published:** 2026-03-16

**Authors:** James Pearson

We thank the reviewer for their insightful comments and suggested revisions. To address each point in order:

 1. The text has been updated to clarify that the listed experiments are those currently within the ESCAPE partners who are themselves invested in open science, and the ESCAPE tools and services are provided for any dark matter experiments invested in open science, with the aim of incorporating more such experiments in future, which will depend on the willingness of such experiments to share their data and process them into an appropriate format.

 2. The text has been updated to have a reduced focus on EDGES, instead expanding the paragraph with more challenges of CDM, such as those mentioned by the reviewer.

 3. The ESCAPE tools have been created to facilitate other researchers (including the associated research infrastructures) to establish such a framework themselves (which is hence beyond the scope of this paper).

 4. The text has been updated to include mention of this, although an in-depth discussion on this topic is beyond the scope of this review.

 5. We appreciate the suggestion, however we feel that it would break the flow of the text and the reader may be confused by the sudden focus on neutrinos, so the text has not been updated to include this.

# Reviewer report: Reviewer response for version 2

**DOI:** 10.21956/openreseurope.23894.r64389

**Published:** 2025-12-29

**Authors:** Helena Garcia Escudero

**Affiliations:** 1 University of California Irvine, Irvine, California, USA

**Report: Overview of the ESCAPE Dark Matter Test Science Project for Astronomers**

**Summary & Context**

**The article presents an overview of the ESCAPE Dark Matter Test Science Project (TSP), describing its goals, tools, and how it seeks to bridge the gap between the particle-physics and astronomy communities in the search for dark matter. The authors note that while dark matter detection efforts come from particle physics (direct detection, indirect detection, collider experiments), the evidence for dark matter’s existence is fundamentally astrophysical or cosmological in nature.**

**The paper argues that a major challenge is that results from particle-physics experiments are often difficult for astronomers to interpret in the context of astrophysical observations, due to different conventions, data formats, and lack of unified tools.ESCAPE aims to address this by providing shared data infrastructure, software, and workflows — via a Virtual Research Environment (VRE), data lake, software catalog, and analysis pipelines — so that different experiments and communities can contribute, compare, and combine their constraints on dark matter in a consistent, reproducible, and interoperable way.**

**The review article is written with the goal of making this project and its tools accessible to astrophysicists, hopefully enabling them to use ESCAPE’s outputs and integrate particle-physics constraints with observational constraints.**

**Is the topic discussed comprehensively, in context of current literature?**

**Strengths:**

- **The article does a good job explaining why a joint dark matter infrastructure is needed: the longstanding separation between particle-physics experiments and astronomical/cosmological observations. This is a well-known challenge in dark matter research.**
- **It outlines clearly the scope of the project: the types of experiments integrated (direct detection, indirect detection, collider experiments) — e.g. DarkSide, ATLAS, KM3NeT, Cherenkov Telescope Array — and describes the infrastructural tools (data lake, VRE, software catalogue) used to unify data/analysis.
 **
- **The authors also note current limitations: the existing TSP tools primarily support particle physics experiments; as of now, astrophysical constraints (e.g. from galaxy rotation curves, lensing, large-scale structure) are not yet integrated.
 **
- **They provide a roadmap / vision for how observational astronomers might contribute, and how the project could help integrate observational data with experimental constraints.
 **

**Limitations / Missing Context:**

- **Because the focus so far has been on particle-physics experiments, the review does not yet cover in detail how astrophysical data (e.g. gravitational lensing, galaxy dynamics, cosmological structure) would be represented within ESCAPE. The authors themselves note this gap.
 **
- **The article does not deeply discuss the limitations, systematic uncertainties, or model-dependence that plague both particle-physics and astrophysical searches. For example: how differences in dark matter halo modeling, astrophysical backgrounds, or assumptions about the dark matter particle (mass, interaction type) might affect the comparability of constraints. A more comprehensive literature review on these challenges would strengthen the discussion.**
- **There is limited discussion about how conflicting results across experiments (or null results) will be reconciled, especially given that many direct- and indirect-detection experiments have produced null results so far. The review might benefit from more context on the existing “state of exclusion limits,” astrophysical vs. experimental tensions, and how ESCAPE aims to handle these.**

**Conclusion on coverage: The topic is discussed well and thoughtfully within the project’s scope. The review provides a clear introduction to the need for integration, outlines the infrastructure developed, and explains how TSP aims to help. However — since this is early-stage infrastructure — the review does not (yet) provide a fully comprehensive discussion of all relevant astrophysical constraints, theoretical uncertainties, or inter-experiment tensions. As a result, the coverage is appropriate for a project-oriented overview, but less so as a definitive review of dark matter constraints in the literature.**

**Are the factual statements correct and well cited?**

**Yes. The review draws on prior work and descriptions of the ESCAPE initiative, the experiments involved, and the challenges faced in dark matter searches. For instance:**

- **The description of ESCAPE’s aims (open science, data lake, VRE, bridging communities) is consistent with prior documentation.**
- **The identification of experiments (direct detection, indirect detection, collider) as part of the TSP scope matches the project’s Indexed scope.**
- **The statement that the existing TSP tools “so far” focus on particle physics experiments and do not yet integrate astrophysical constraints is explicitly acknowledged.**

**Where the authors note limitations or gaps (e.g. lack of astrophysical data integration), they do not overstate — which is scientifically honest.**

**Because this is a short review article (10 pages, no figures), the authors do not make sweeping claims beyond the status of the TSP and its goals; hence, the factual statements are modest and correctly supported.**

**Thus: factual correctness and citation support — yes.**

**Is the review written in accessible language?**

**Overall — yes, the review is reasonably accessible for its intended audience (astronomers / astrophysics researchers), while remaining scientifically appropriate. There are a few points for improvement.**

**Positives:**

- **The writing is clear and directed toward an astronomical audience (not necessarily particle-physics experts), which was the explicit goal. The introduction frames the historical separation between astrophysical evidence and particle-physics experiments in understandable terms.**
- **Technical aspects (data infrastructure, VRE, examples of experiments) are described in conceptual terms, without unnecessary jargon, which makes the article approachable for astronomers or non-experts.**
- **The structure is logical: explanation of the problem, introduction of ESCAPE, description of tools and experiments, discussion of (future) use by astronomers.**

**Areas for improvement:**

- **Because the article necessarily involves several technical concepts (collider vs direct/indirect detection; data lakes; FAIR data; Virtual Research Environments), some readers without prior familiarity with computational infrastructure may find parts less accessible. Perhaps a brief “Glossary” or more contextualization for non-particle physicists could help.**
- **The article assumes some familiarity with common dark matter search methods. For example, it lists several experiments by name; someone entirely new to dark matter research might struggle to place their significance.**

**Are the conclusions drawn appropriate in the context of current research literature?**

**Yes — the conclusions are modest, realistic, and well justified:**

- **The authors conclude that the ESCAPE TSP provides a promising framework for cross-community collaboration by providing data and software infrastructure, which may help unify dark matter constraints from diverse experiments. This is supported by the description of the VRE, data lake, and the experimental scope.**
- **They also recognize current limitations: that astrophysical constraints and observational data are not yet integrated, and that further work is needed to expand tools to cover the astronomy side.**
- **The recommendation that astrophysicists engage with ESCAPE — using its tools, contributing observational constraints, and helping interpret experimental results in astrophysical context — is reasonable and aligns with the broader need for interdisciplinary cooperation in dark matter research.**

**Given the complexity and uncertainty inherent in dark matter searches, drawing bold claims (e.g. “we will find dark matter soon”) would be inappropriate — but the authors avoid such overreach. Their conclusions remain grounded in what ESCAPE currently offers, and what it could enable in the future. That caution is appropriate.**

**Typos and Terminology**

- **In the introduction, Standard Model is mentioned for the first time, but the abbreviation is not introduced at that point. The authors later use the acronym “SM,” but it is not originally defined. I recommend introducing it upon first use, for example: “Standard Model (SM)” and replace SM consistently within the text.**

**Overall Assessment and Recommendations**

**Overall, this review article is a valuable, timely, and well-crafted contribution. It addresses a real, persistent problem in dark matter research — the fragmentation between experimental and observational communities — and describes a concrete solution: shared infrastructure under ESCAPE. For astronomers or astrophysicists who have not closely followed the particle-physics side of dark matter searches, this paper serves as an excellent entry point. It also provides value for more knowledgeable experts by outlining the emerging ecosystem of shared data infrastructures, analysis tools, and cross-experiment pipelines, giving them a clear view of how their work can integrate with broader multi-messenger efforts and how upcoming open-science platforms may streamline collaborative, cross-disciplinary dark matter research.**

**For future improvements or next-phase work, I recommend:**

- **Integrating observational astrophysics data (e.g. from galaxy dynamics, gravitational lensing, cosmological surveys) into the ESCAPE tools, so that astronomers can directly use those data to compare with experimental constraints.**
- **Providing tutorials, documentation, or user-friendly interfaces for the VRE / data to lower the barrier for astronomers unfamiliar with particle-physics workflows.**
- **Expanding the review to discuss limitations, model dependencies, and uncertainties in both experimental and observational dark matter constraints — which could help users critically interpret combined results.**

**Offering case studies or example analyses that combine experimental (e.g. direct detection) constraints with astrophysical observations, to demonstrate how ESCAPE tools can be used in practice.**

Is the review written in accessible language?

Yes

Are all factual statements correct and adequately supported by citations?

Yes

Are the conclusions drawn appropriate in the context of the current research literature?

Yes

Is the topic of the review discussed comprehensively in the context of the current literature?

Yes

Reviewer Expertise:

Cosmology and astroparticle physics

I confirm that I have read this submission and believe that I have an appropriate level of expertise to confirm that it is of an acceptable scientific standard.

# Response

**Published:** 2026-03-16

**Authors:** James Pearson

We thank the reviewer for their insightful comments and suggested revisions. To address each point in order: 1. Limitations / Missing Context:

 a) Given the widely different astrophysical observations, the data may vary substantially and hence how the data would be represented in ESCAPE will require close collaboration with the specific astrophysical communities, which the leads of the Dark Matter Test Science Project encourage.

 b & c) Such a comprehensive literature review over astrophysics and particle physics dark matter searches is beyond the scope of this paper - apt discussions of exclusion limits, tensions and limitations already feature in the more comprehensive reviews cited in the Introduction. Meanwhile, this paper aims to focus on the DM TSP project while providing some examples of such searches for integration with ESCAPE tools. Through discussion on the needs of each community, ESCAPE tools are aimed at facilitating researchers to handle such tensions etc., rather than the ESCAPE project handling them itself.

 2. Accessibility:

 a) The VRE & data lake has been expanded upon in the text. FAIR principles are commonly employed and fairly self-explanatory given the abbreviation and reference, and each particle experiment type is explained at the start of the relevant subsection.

 b) The text has been updated to clarify that the listed experiments are those currently within the ESCAPE partners who are themselves invested in open science.

 3. Typos: This has now been corrected.

 4. Future improvements or next-phase work: We appreciate the detailed list of ideas for where to take the work next, though here we leave that to future work.

# Reviewer report: Reviewer response for version 2

**DOI:** 10.21956/openreseurope.23894.r65288

**Published:** 2025-12-19

**Authors:** Eugene Oks

**Affiliations:** 1 Auburn University, Auburn, AL, USA

The paper has been improved, but it still has significant flaws, as follows.

    First, the authors refer to Singh et al (2022) observation at SARAS 3 as not confirming the anomalous 21 cm absorption signal observed at the EDGES by Bowman et al (2018). However, Prof. Bowman (the Head of the EDGES team) in his private communication to me shared with me the following *concerns with the Singh et al paper*.

- 1. The limited amount of data which causes *low sensitivity and reduces options for testing for instrumental effects.*
- 2. The narrow bandwidth that makes the 21cm *parameter estimation weaker and less robust.*
- 3. The lack of assessment of *systematic errors* in their parameter estimation.    

 

 Prof. Bowman added: “These experiments are extremely challenging.  As we noted in the 2018 paper, we made many tests of the instrument and analysis before reporting our evidence of a detection.”

    Further problems with Singh et al (2022) observation at SARAS 3 are the following.

- At the SARAS 3, there was some *unwanted contribution* from receiver noise and thermal emission from water beneath the antenna, the antenna being floated on a large body of water (on lakes in Southern India).
- The *noise level* at the SARAS 3 was 213 mK, which was several times higher than the noise level of 87 mK at the EDGES.
- *Radio Frequency Interference (RFI):* At the SARAS 3 observing sites in India, substantial RFI was encountered up to 55 MHz and the spectrum was also unusable above 85 MHz. In distinction, in Australia (where the EDGES is located), there are no licensed digital TV transmitters below 174 MHz, what allowed Bowman et al (2018) to perform observations in the broader range of 50 – 100 MHz.

   For all of the above reasons, Singh et al (2022) observation at the SARAS 3 are **unreliable **compared to Bowman et al (2018) observation at the EDGES. Last but not least: according to Abdurashidova et al paper of 2022 (Astrophys. J. 924, 51), the latest observations at the Hydrogen Epoch Reionization Array (HERA) do **not disprove** the results from EDGES of 2018.

    Second, the authors refer to alternative explanations of the anomalous 21 cm signal, citing Bevins et al (2022) paper and references therein. So, let us briefly analyze the alternative explanations.

1. A possible cooling of baryons by some exotic dark matter particles of the charge of the million times smaller than the electron charge, as hypothesized in paper by Muñoz & Loeb of 2018 (Nature 557, 684) and Liu et al paper of 2019 (Phys. Rev. D, 100, 123011). However, the *hypothetical* exotic dark matter particles of the charge of the million times smaller than the electron charge were never discovered experimentally. Besides, Liu et al (2019) estimated that if there would be charged dark matter particles, they can only constitute ∼ 10 ^–8 ^of the total dark matter energy density.
2. Another alternative explanation requires an additional *hypothetical *radio background suggested by Feng & Holder (2018, Astrophys. J. 858, L17), Ewall-Wice et al (2018, Astrophys. J. 868, 63), Fialkov & Barkana (2019, MNRAS 486, 1763), and Reis, Fialkov & Barkana (2020, MNRAS 499, 5993). However, Sharma showed (already in 2018, MNRAS 481, L6) and Cang et al reconfirmed (in 2024, arXiv: 2411.08134.v1) that an additional radio background **cannot** explain the observed absorption signal.

   Therefore, the most plausible explanation of the anomalous 21 cm signal is the one suggested by Barkana (2018, Nature, 555, 71) and reconfirmed by McGaugh (2018, Research Notes Amer. Astron. Soc. 2, 37): baryonic dark matter particles cooled down the primordial hydrogen gas by collisions. In paper published in Research in Astronomy and Astrophysics (2020, 20, 109) it was shown that if the unspecified (in the above papers by Barkana and by McGaugh) baryonic dark matter particles were the Second Flavor of Hydrogen Atoms (SFHA), then the anomalous 21 cm signal can be explained not only qualitatively, but also quantitatively.

    Theoretically, the SFHA results from the second solution of the standard Dirac equation for hydrogen atoms – the solution that became legitimate for all S-states (i.e., the states of the zero angular momentum) after the allowance for the finite size of protons and for the actual experimental charge density distribution inside protons. Due to the selection rules of quantum mechanics, the SFHA by virtue of having only the S-states, cannot emit or absorb the electromagnetic radiation: the SFHA remains *dark*. Most significantly, the SFHA is not just a theoretical construct – the actual existence of the SFHA is evidenced by three different types of atomic experiments: these experiments revealed huge (up to several orders of magnitude) discrepancies with the corresponding previous theories and the SFHA-based explanations completely removed these huge discrepancies, while alternative explanations were never provided. Last but not least: the SFHA-based explanation completely resolved the long-standing puzzle of the neutron lifetime; so, the SFHA does exist – for details see, e.g., papers published in Int. J. Mod. Phys. D 2025, 34, 2550008, in Nuclear Phys. B 2025, 1014, 116879, in Symmetry 2025, 17, 517, as well as the review published in New Astronomy Reviews 2023, 96, 101673.

    The most important: in distinction to all attempted alternative explanations of the anomalous 21 cm signal, the theory behind the SFHA *does not make any assumptions* and, being based on the standard Dirac equation, is *within the Standard Model.* Therefore, it is favored by the *Occam Razor principle* stating that when several theories compete, the one that makes the least assumptions is the most probable to correspond to reality. Thus, the *existing* SFHA is more favorable than all alternative, exotic explanations of the anomalous 21 cm signal.

    Because of the above significant flaws in the revised paper, I still cannot recommend it for indexing.

Is the review written in accessible language?

Yes

Are all factual statements correct and adequately supported by citations?

Partly

Are the conclusions drawn appropriate in the context of the current research literature?

Yes

Is the topic of the review discussed comprehensively in the context of the current literature?

Partly

Reviewer Expertise:

Astrophysics; Particle Physics

I confirm that I have read this submission and believe that I have an appropriate level of expertise to state that I do not consider it to be of an acceptable scientific standard, for reasons outlined above.

# Response

**Published:** 2026-03-16

**Authors:** James Pearson

We thank the reviewer for their insightful comments and suggested revisions. While we appreciate the detailed response, we do not believe the paper should be significantly modified on the basis of unpublished private communications between third parties. In agreement with comments from other reviewers, we have expanded the discussion on problems with lambda-CDM and their alternate explanations and slightly reduced the focus specifically on the EDGES signal as EDGES (and indeed a fully in-depth review of dark matter candidates) is not the focus of this work – such comprehensive discussions of the EDGES signal and the limitations of all other works would be best left to another review.

# Reviewer report: Reviewer response for version 1

**DOI:** 10.21956/openreseurope.23379.r63718

**Published:** 2025-11-13

**Authors:** Eugene Oks

**Affiliations:** 1 Auburn University, Auburn, AL, USA

  The paper provides a useful overview of the tools for the particle physics community involved in the experimental searches for dark matter. The authors encourage astronomers to utilize the ESCAPE services within Dark Matter Test Science Project.

    However, the paper has significant flaws, as follows.

    1. From astrophysical observations (Planck Collaboration) follows that in the Universe there are two types of Dark Matter (DM): baryonic DM and non-baryonic DM – baryonic DM is in the ratio 1 to 5 to non-baryonic DM (see, e.g., review by Arbey and Mahmoudy, Progr. Part. Nucl. Phys., 119 (2021) 103865). Therefore, the authors should clearly state in Introduction the existence of these two types of DM, both definitely existing, as shown by the astrophysical observations – otherwise the readers would be confused from the very beginning of the paper.

    2. Out of the major astrophysical observations, causing to resort to DM, the authors mention only the flattening of the rotation curves of galaxies and gravitational microlensing. However, there is also the third type: the observation of the anomalous absorption in the redshifted 21 cm line from the early Universe (Bowman et al, Nature 555 (2018) 67) – the absorption signal was found to be 2 to 3 times stronger than predicted by the standard cosmology. This meant that the primordial hydrogen gas was significantly cooler than expected from the standard cosmology.

 The consistent, noncontroversial explanation of this anomalous absorption required baryonic DM that cooled the hydrogen gas by collisions (Barkana, Nature 555 (2018) 71; McGaugh, Res. Notes Amer. Astron. Soc. 2 (2018) 37). 

    3. While referring to reviews on DM in peer-reviewed journals, the authors cite only relatively old reviews indexed between 2002 and 2020. However, there are more recent reviews on DM indexed in the peer-reviewed journal of the tremendous impact factor 24.8: New Astronomy Reviews 96 (2023) 101673; 93 (2021) 101632.

    Thus, the paper requires a major revision.

Is the review written in accessible language?

Yes

Are all factual statements correct and adequately supported by citations?

Partly

Are the conclusions drawn appropriate in the context of the current research literature?

Yes

Is the topic of the review discussed comprehensively in the context of the current literature?

Partly

Reviewer Expertise:

Astrophysics; Particle Physics

I confirm that I have read this submission and believe that I have an appropriate level of expertise to state that I do not consider it to be of an acceptable scientific standard, for reasons outlined above.

# Response

**Published:** 2025-11-28

**Authors:** James Pearson

We thank the reviewer for their insightful comments and suggested reviews. To address each point in order:

 1. As a clarification, the suggested review generally refers to dark matter as being separate from baryonic matter, with the ratio of all baryonic matter to cold dark matter being around 1:5 according to the quoted Planck18 results. Through works by e.g. de Graaff et al. (2019), about 30 percent of the baryon budget can be accounted for, and so the rest could be regarded as 'baryonic dark matter', giving a ratio of baryonic-to-non-baryonic dark matter of roughly 1:7.6. Regardless, the first paragraph of the Introduction has been updated to clarify the existence of both baryonic and non-baryonic dark matter and the focus of this work on the latter.

 2. Additional text has been added to the paper regarding the anomalous absorption of the redshifted 21cm line in data from the Experiment to Detect the Global Epoch of Reionization Signature (EDGES; Bowman et al. 2018). We mention that this could be evidence for baryonic dark matter (Barkana 2018; McGaugh 2018), however we also highlight that this detection has not been confirmed, such as the non-detection by the Shaped Antenna measurement of the background RAdio Spectrum 3 experiment (SARAS 3; Singh et al. 2022), with other works having sought alternative explanations (see e.g. Bevins et al. 2022 and references therein). It is also worth noting that the CMB gives an approximately 100-sigma detection of collisionless (i.e. non-baryonic) dark matter (Planck Collaboration 2018; Arbey & Mahmoudi 2021; Turner 2025), providing concrete evidence of non-baryonic dark matter - this has also been added to the Introduction. Also as a clarification, while the lack of gravitational microlensing of MACHOS have ruled out some mass ranges for baryonic dark matter, gravitational microlensing itself does not provide any evidence for non-baryonic dark matter; rather, it is strong and weak gravitational lensing on cosmological scales that provides this evidence and hence what we focus on here.

 3. We have included references to more recent reviews, including the suggested Oks (2021) paper, Arbey & Mahmoudi (2021), and a recent review by Cirelli et al. (2024).
